# Dietary Tocotrienol/*γ*-Cyclodextrin Complex Increases Mitochondrial Membrane Potential and ATP Concentrations in the Brains of Aged Mice

**DOI:** 10.1155/2015/789710

**Published:** 2015-08-02

**Authors:** Anke Schloesser, Tuba Esatbeyoglu, Stefanie Piegholdt, Janina Dose, Naoko Ikuta, Hinako Okamoto, Yoshiyuki Ishida, Keiji Terao, Seiichi Matsugo, Gerald Rimbach

**Affiliations:** ^1^Institute of Human Nutrition and Food Science, University of Kiel, Hermann-Rodewald-Straße 6, 24118 Kiel, Germany; ^2^Graduate School of Medicine, Kobe University, 7-5-1, Kusunoki-cho, Chuo-ku, Kobe 650-0017, Japan; ^3^CycloChem Bio Co., Ltd., KIBC, 5-5-2 Minatojima-minamimachi, Chuo-ku, Kobe 650-0047, Japan; ^4^School of Natural System, Kanazawa University, Kakuma-machi, Kanazawa 920-1192, Japan

## Abstract

Brain aging is accompanied by a decrease in mitochondrial function. In vitro studies suggest that tocotrienols, including *γ*- and *δ*-tocotrienol (T3), may exhibit neuroprotective properties. However, little is known about the effect of dietary T3 on mitochondrial function in vivo. In this study, we monitored the effect of a dietary T3/*γ*-cyclodextrin complex (T3CD) on mitochondrial membrane potential and ATP levels in the brain of 21-month-old mice. Mice were fed either a control diet or a diet enriched with T3CD providing 100 mg T3 per kg diet for 6 months. Dietary T3CD significantly increased mitochondrial membrane potential and ATP levels compared to those of controls. The increase in MMP and ATP due to dietary T3CD was accompanied by an increase in the protein levels of the mitochondrial transcription factor A (TFAM). Furthermore, dietary T3CD slightly increased the mRNA levels of superoxide dismutase, *γ*-glutamyl cysteinyl synthetase, and heme oxygenase 1 in the brain. Overall, the present data suggest that T3CD increases TFAM, mitochondrial membrane potential, and ATP synthesis in the brains of aged mice.

## 1. Introduction

**Figure 1:**
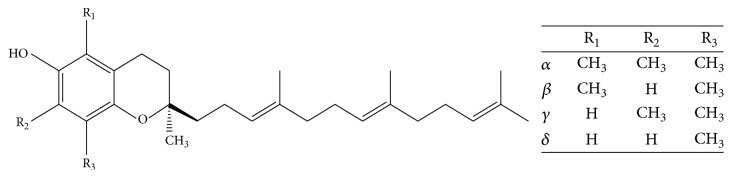
The chemical structure of tocotrienol isoforms.

Tocotrienols are plant derived bioactives. Tocotrienols occur in nature in four different isoforms: *α*-, *β*-, *γ*-, and *δ*-tocotrienol (T3). The *α*-form of tocotrienol contains 3 methyl groups, the *β*- and *γ*-forms contain two groups, and the *δ*-form contains only one methyl group at the chromanol ring [1]. Important sources of *α*-tocotrienol are palm oil, rice bran, and barley, whereas annatto contains significant amounts of *γ*- (10%) and *δ*-tocotrienol (90%). The chemical structure of the tocotrienol isoforms is illustrated in Figure 1.

Cyclodextrins are widely used to improve the solubility and stability of lipophilic molecules including tocotrienols [2]. It has been recently shown that complexation of annatto-derived tocotrienols with *γ*-cyclodextrin (CD) significantly improved tocotrienol bioavailability in rats [3] and mice [2] by enhancing intestinal absorption. Furthermore, complexation of T3 with CD significantly prolonged the life span of the model organism,* Caenorhabditis elegans* [4].

Brain aging is accompanied by decreased mitochondrial function, increased oxidative stress, decreased proteasomal activity, and the accumulation of cytotoxic amyloid beta peptides. TFAM and PGC1*α* are key transcription factors that control mitochondrial biogenesis. TFAM activates the duplication of mitochondrial DNA molecules, thereby orchestrating mitochondrial bioenergetics and ATP production [5]. Mitochondrial dysfunction may result in elevated production of reactive oxygen species. In addition, an age-related loss of endogenous antioxidant defense mechanisms such as superoxide dismutase (Sod), heme oxygenase 1 (Hmox1), and *γ*-glutamyl cysteinyl synthetase (Gclm) accelerates oxidative stress in the brain. Similarly, during the aging process, amyloid beta peptides may enter mitochondria and disrupt mitochondrial function [6]. It has also been demonstrated that proteasomal activity decreases in the aging brain; thus, the cellular ability to degrade oxidized proteins may be impaired [7]. Collectively, all of these processes are interrelated and contribute to an accelerated brain aging phenotype.

There is increasing experimental evidence that T3 prevents cellular senescence [8]. In fact, the age-dependent decrease in the number of brain cells may be slowed down or partly counteracted by T3-rich nutraceuticals [9]. It has been suggested that dietary T3 reaches the brain [10] and exhibits neuroprotective properties [11–14]. However, data on neuroprotection, in the context of mitochondrial function, due to tocotrienols, are scarce. Therefore, in this study, we investigated the effect of annatto-derived dietary tocotrienols complexed with *γ*-cyclodextrin on mitochondrial membrane potential and ATP concentrations in the brains of aged mice.

## 2. Materials and Methods

### 2.1. Mice and Diet

Animal studies were performed according to German regulations of animal welfare and with the permission of the appropriate local authorities.

Male C57BL/6J mice were purchased from Taconic Europe A/S (Ry, Denmark) at the age of 15 months. The mice were housed individually in Makrolon cages under controlled environmental conditions (22–24°C, 45–55% relative humidity, and 12 h light/dark cycle) with free access to diet and tap water. The experimental diet (Table 1) was a purified semisynthetic energy dense high fat and high sugar Western-type diet purchased from Ssniff (TD 88137 modified, Soest, Germany). For the first 2 weeks, all mice were fed the Western-type diet ad libitum without tocotrienol/*γ*-cyclodextrin. Subsequently, mice were randomly assigned to body weight-matched diet groups, namely, the control (*n* = 6) and the tocotrienol/*γ*-cyclodextrin (T3CD, *n* = 8) supplemented group. Annatto-derived tocotrienol/*γ*-cyclodextrin (Cyclochem Bio Co., Ltd., Kobe, Japan) was supplemented at 369 mg/kg (providing 100 mg T3/kg diet) in the diet for 24 weeks. Diets were supplemented with 20 mg/kg diet *α*-tocopherol.

Feed intake and body weight were determined on a weekly basis. At week 20, the energy expenditure was measured using a TSE PhenoMaster (TSE Systems GmbH, Bad Homburg, Germany) as previously described [13]. We lost one mouse in the control group during the supplementation period. At the end of the trial, the mice were killed by cervical dislocation, and the brains were immediately removed and dissected into hemispheres. One entire hemisphere was used for the preparation of dissociated brain cells, and the other one was snap-frozen in liquid nitrogen and stored at −80°C until further analysis (except for samples used for RNA isolation, which were placed in RNAlater (Qiagen, Hilden, Germany) and stored at −20°C).

### 2.2. Preparation of Dissociated Brain Cells

The preparation of dissociated brain cells was performed according to [15] with some modifications. The cerebellum was excluded, and the remaining tissue was quickly dissected on ice, washed, and then minced in 2 mL of medium I (138 mM NaCl, 5.4 mM KCl, 0.17 mM Na_2_HPO_4_, 0.22 mM KH_2_PO_4_, 5.5 mM glucose, and 58.4 mM sucrose; pH 7.35) with a scalpel. The brain cells were further dissociated first by filtration through a crude filter (200 *μ*m) using a Pasteur pipette and later through a fine filter (100 *μ*m). The cell suspension was then washed twice in medium II (110 mM NaCl, 5.3 mM KCl, 1.8 mM CaCl_2_, 1 mM MgCl_2_, 25 mM glucose, 70 mM sucrose, and 20 mM 4-(2-hydroxyethyl)piperazine-1-ethanesulfonic acid (HEPES); pH 7.4). After a final centrifugation step (1000 ×g, 5 min, 4°C), the cell pellet was resuspended in 5 mL Dulbecco's modified Eagle's medium (DMEM, Gibco, Life Technologies, Germany) and distributed 50 *μ*L/well in white 96-well plates for adenosine triphosphate (ATP) measurements and 250 *μ*L/well in 24-well plates for mitochondrial membrane potential (MMP) measurements. The plated dissociated brain cells were incubated at 37°C in 5% CO_2_ for 3 h prior to measurements. ATP concentrations and MMP are expressed as fluorescence per milligram protein, and the protein content was determined using the BioRad DC Protein Assay (BioRad, Munich, Germany).

### 2.3. Measurement of Mitochondrial Membrane Potential (MMP) and Adenosine Triphosphate (ATP) Concentrations

Basal MMP was determined using the Rhodamine 123 (Sigma-Aldrich, Steinheim, Germany) fluorescence dye as previously described [9]. Basal ATP concentrations were measured using the ViaLight Plus Bioluminescence Kit (Lonza, Walkersville, USA) according to [15].

### 2.4. Cerebral Proteasome Activity

Proteasomal activity in the brain tissue was measured according to [16] with some modifications. Up to 10 mg brain tissue was homogenized 1 : 10 in ice-cold lysis buffer (20 mM Tris-HCl, 10% glycerol, 0.5 mM EDTA, 0.5% Nonidet P-40, 5 mM MgCl_2_, 1 mM dithiothreitol, and 1 mM adenosine triphosphate; pH 7.8) for 2 × 2 min at 25 Hz with the TissueLyser II (Qiagen, Hilden, Germany), incubated on ice for 30 min, and centrifuged (15,700 ×g; 10 min; 4°C) to maintain the supernatant for further analysis. In addition, 20 *μ*g protein of each sample was incubated with 185 *μ*L reaction buffer (20 mM Tric-HCl, 5 mM MgCl_2_, 1 mM dithiothreitol, and 1 mM adenosine triphosphate; pH 7.8) and 5 *μ*L of the fluorescent substrate N-succinyl-leucineleucine-valine-tyrosine-7-amino-4-methylcoumarin (Suc-LLVY-AMC, Enzo Life Sciences, Lörrach, Germany). The initially quenched fluorescence signal was measured on a Tecan infinite F200 plate reader (Tecan, Grödig, Austria) at 360 and 465 nm excitation and emission wavelengths, respectively. The calculation of the proteasomal activity was based on an external standard curve.

### 2.5. Gene Expression Using qRT-PCR

RNA isolation and qRT-PCR were conducted as previously described [15]. Primer sequences are given in Table 2. Relative mRNA levels were calculated with an external standard curve and were related to housekeeping gene expression (Actb). The mean value of expression in the control group was set to an arbitrary unit of 1.

### 2.6. Western Blotting

Protein expression was determined in cytosolic lysates prepared from brain tissue except for BACE1, which was detected in whole cell lysates. Samples (40–60 *μ*g protein) were mixed with loading buffer (0.5 M Tris-HCl, 8% glycerol, 1.6% sodium dodecyl sulfate, 0.001% brome phenol blue, and 5% *β*-mercaptoethanol), denatured at 95°C for 5 min, and loaded on Criterion TGX Stain-Free Precast gels (BioRad, Munich, Germany) for separation by SDS–PAGE. Protein fluorescence was activated by UV-exposition for 5 min before transfer onto a PVDF membrane using the Trans-Blot Turbo System (BioRad, Munich, Germany). The target proteins were identified using respective primary (Table 3) and secondary antibodies (Santa Cruz Biotechnology, Heidelberg, Germany) as previously described [16].

### 2.7. Statistical Analysis

All presented data are expressed as the means ± SEM. Statistical analysis was based on Mann-Whitney *U* test. Values of *p* < 0.05 were considered statistically significant and are indicated with asterisks (∗). The statistical analysis was performed using PASW Statistics 18 (IBM, Chicago, IL, USA).

## 3. Results

There were no significant differences in feed intake, final body weight, or energy expenditure between the control mice and the mice receiving the T3CD enriched diet as summarized in Table 4.

**Figure 2:**
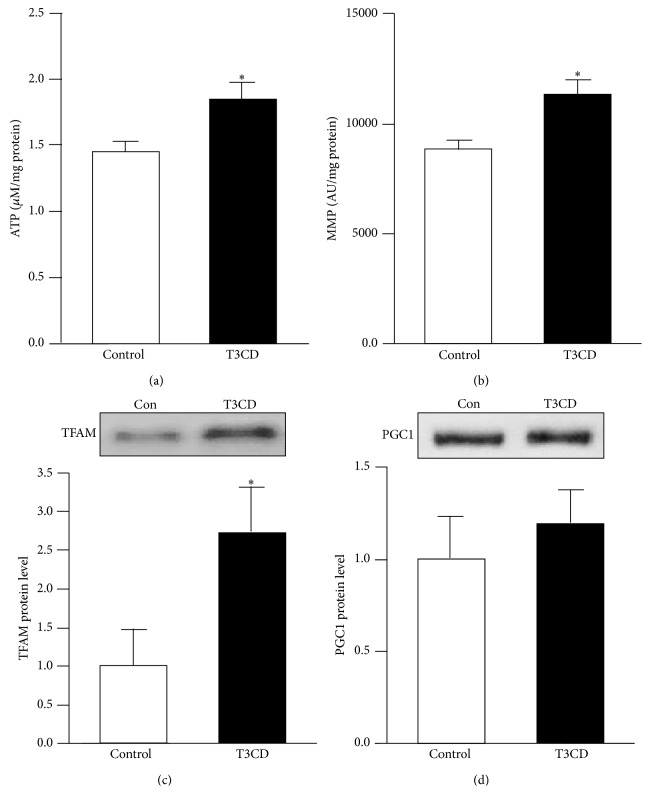
Basal adenosine triphosphate (ATP) concentration, mitochondrial membrane potential (MMP), and TFAM protein levels in mouse brain were elevated by tocotrienol/*γ*-cyclodextrin supplementation. (a) Basal ATP [*μ*M/mg protein] and (b) MMP [AU/mg protein] levels were measured in dissociated brain cells that were freshly isolated from the mice fed either a control diet or a diet supplemented with tocotrienol/*γ*-cyclodextrin (T3CD) complex. (c) TFAM and (d) PGC1 protein levels were determined by Western blotting and subsequent densitometric analysis of target bands. Target protein expression was related to the total protein fluorescence transferred to the PVDF membrane. Representative blots from one of 5–8 animals per groups are shown. Values are the means + SEM from 5 to 8 animals per group. The asterisks indicate a significant difference (*p* < 0.05) between the groups.

To assess brain bioenergetics, we determined the ATP concentrations and mitochondrial membrane potential in the mice. The ATP concentrations in dissociated brain cells were significantly higher in the T3CD-fed mice than in those of the controls (Figure 2(a)). Consistent with higher brain ATP levels, we observed a significantly higher membrane potential in the brains of the mice fed the T3CD enriched diet (Figure 2(b)). Under the conditions investigated, T3CD versus control mice exhibited significantly higher TFAM protein levels (Figure 2(c)). However, the protein levels of PGC1*α* were similar between the groups as shown in Figure 2(d).

**Figure 3:**
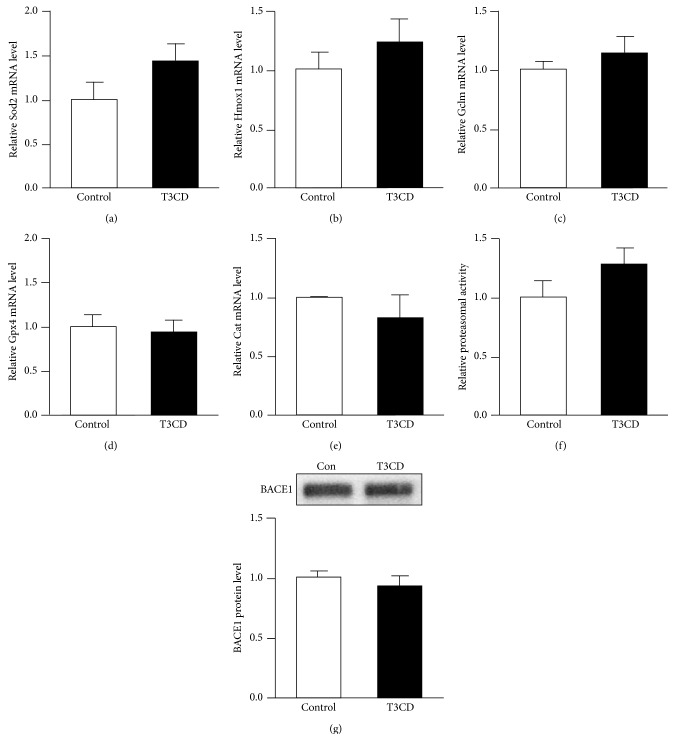
Effect of tocotrienol/*γ*-cyclodextrin supplementation on the mRNA levels of Sod2, Hmox1, Gclm, Gpx4, and Cat, proteasomal activity, and the BACE1 protein levels in mouse brain. Relative mRNA levels of (a) Sod2, (b) Hmox1, (c) Gclm, (d) Gpx4, and (e) Cat were determined by qRT-PCR and were related to the mean of the housekeeping gene expression. (f) Proteasomal activity was measured in the brain tissue by releasing the initially quenched fluorescence signal of the substrate through cleavage by the specific proteasome site. (g) BACE1 protein levels were determined by Western blotting and subsequent densitometric analysis of target bands. Target protein expression was related to the total protein fluorescence transferred to the PVDF membrane. Representative blots from one of 5–8 animals per group are shown. Values are the means + SEM from 5 to 8 animals per group.

Dietary T3CD slightly increased (44% increase) Sod2 (Figure 3(a)) compared to that of controls. We also observed slightly higher Hmox1 (22% increase) and Gclm (14% increase) levels as well as higher proteasomal activity (28% increase) in the brains of mice fed the T3CD enriched diet; however, the differences did not reach statistical significance (Figures 3(b), 3(c), and 3(f)). Brain Gpx4 and Cat mRNA concentrations (Figures 3(d) and 3(e)) remained unchanged by the different dietary treatments.

Furthermore, the BACE1 protein concentrations were measured in this study in response to dietary T3CD. However, there were no significant differences in the BACE1 protein concentrations between the T3CD and control mice (Figure 3(g)). In addition, gamma secretase protein concentrations (data not shown) remained unchanged by the different dietary treatments.

## 4. Discussion

It is well documented in the literature that mitochondrial biogenesis is impaired in aging [5]. Therefore, in this study, we included middle-aged mice (21 months old by the end of the study) in our feeding trial.

Differences in feed intake may affect mitochondrial function. In fact, caloric restriction has been reported to affect mitochondrial biogenesis and specific TFAM binding to mitochondrial DNA in laboratory rodents [17]. Therefore, over the 6-month experiment, we continuously monitored feed intake, which was not significantly different between the controls and the T3CD-fed mice. Accordingly, no differences in body weight or energy expenditure were evident between the groups.

In the present study, we found higher ATP concentrations and higher mitochondrial membrane potential in the murine brain due to dietary T3CD. Thus T3CD may partly counteract an aging brain phenotype in terms of mitochondrial function. We suggest that higher ATP concentrations and MMP may be related to higher TFAM concentrations. Studies in transgenic mice indicate that TFAM is a direct regulator of mtDNA copy number [18], thereby driving mitochondrial biogenesis. Our in vivo data in mice are consistent with in vitro studies demonstrating that T3 improves mitochondrial respiration, coupling, and membrane potential and maintains oxidative phosphorylation and ATP levels in cultured cells [19].

PGC1*α* is a master switch in energy metabolism. A decrease in PGC1*α* may impair mitochondrial respiratory capacity, thereby increasing the production of reactive oxygen species [20]. Unlike the reported effects of coenzyme Q10, lipoic acid [20], and tocopherol [21], we did not observe an increase in PGC1*α* in T3CD treated animals. Thus, the increase in mitochondrial membrane potential and ATP concentrations in the brain of our aged mice due to T3CD was possibly not directly mediated via a PGC1*α*-dependent signal transduction pathway. Possibly, other key regulators of mitochondrial biogenesis (e.g., sirtuins, endothelial nitric oxide synthase, and mitofusin) may be involved in the tocotrienol-mediated increase in mitochondrial membrane potential and ATP concentration, which warrants further investigation.

Superoxide dismutase, *γ*-glutamyl cysteinyl synthetase, and heme oxygenase 1 are Nrf2 target genes. Studies in laboratory mice have revealed that the induction of the Nrf2-driven antioxidant response confers neuroprotection during mitochondrial stress in vivo [22]. Furthermore, it has been shown in cultured cells that *γ*- and *δ*-tocotrienol may induce Nrf2-dependent signal transduction pathways [23, 24]. In addition, there is crosstalk between Nrf2 and the proteasome [25]. Therefore, we measured Sod2, Hmox1, and Gclm gene expression as well as proteasomal activity in mice brain in response to dietary T3CD. Under the conditions investigated, only a moderate increase in Sod2, Hmox1, Gclm, and proteasomal activity due dietary T3CD was observed. Further studies should address the question of whether higher dietary T3CD concentrations lead to a more pronounced induction of Nrf2 and its target genes.

We observed no changes in BACE1 in response to dietary T3CD. Similar results were observed in our previous studies in mice [26] and rats [27] that were fed diets containing different concentrations of *α*-tocopherol; thus tocopherols and tocotrienols seem to have little to no effect on BACE1 expression in laboratory rodents.

The present study has several limitations. First, we did not measure T3 transfer into the brain because of the limited availability of brain tissue, all of which was used for ATP, MMP, Western blot, and RT-PCR analyses. Brain T3 concentrations have been reported elsewhere [28]. However, we measured the transfer of *δ*-T3 into white adipose tissue. In the T3CD-fed mice, we analyzed 16.0 ± 1.90 nmol T3 per g fresh matter indicating sufficient absorption and tissue distribution of tocotrienols provided as dietary T3CD. Furthermore, we did not conduct brain imaging [29], behavioral and cognitive testing in response to dietary T3 as previously described [28]. Only few rodent species spontaneously develop the cognitive, behavioral, and neuropathological symptoms of age-related diseases [30]. Thus, such tests should be conducted in mice exhibiting a pronounced brain aging phenotype (e.g., transgenic mouse models of Alzheimer's disease).

## 5. Conclusions

In conclusion, our results suggest that complexation of annatto-derived tocotrienols with *γ*-cyclodextrin significantly enhances mitochondrial membrane potential and ATP concentrations in the brains of aged mice. Further in vivo studies are required to elucidate whether dietary T3CD affects behavior and cognitive function in animal models of age-related brain disorders.

## Figures and Tables

**Table 1:** Composition of the experimental diets.

	Control	T3CD
Tocotrienol/*γ*-cyclodextrin	—	369 mg/kg^∗^
Crude protein	17.1%	17.1%
Crude fat	21.2%	21.2%
Crude fiber	5.0%	5.0%
Crude ash	4.5%	4.5%
Nitrogen free extracts	49.3%	49.3%
Starch	14.5%	14.5%
Sugar	32.8%	32.8%
Cholesterol	0.2%	0.2%
Vitamin E	20 mg/kg	20 mg/kg

Metabolizable energy	19.1 MJ/kg	19.1 MJ/kg

^∗^Providing 100 mg T3 per kg diet.

**Table 2:** Primer sequences and annealing temperatures used for qRT-PCR analyses in murine brain tissue.

Gene symbol	Gene name	Forward 5′-3′	Reverse 3′-5′	Annealing temperature
Actb	Actin, beta	GACAGGATGCAGAAGAGATTACT	TGATCCACATCTGCTGGAAGGT	55°C
Cat	Catalase	GGAGCAGGTGCTTTTGGATA	CTGACTCTCCAGCGACTGTG	55°C
Gclm	Glutamate-cysteine ligase, modifier subunit	TCCCATGCAGTGGAGAAGAT	AGCTGTGCAACTCCAAGGAC	57°C
Gpx4	Glutathione peroxidase 4	ATGAAAGTCCAGCCCAAGG	CGGCAGGTCCTTCTCTATCA	59°C
Hmox1	Heme oxygenase 1	GAGCCTGAATCGAGCAGAAC	AGCCTTCTCTGGACACCTGA	59°C
Sod2	Superoxide dismutase 2, mitochondrial	GCCTGCTCTAATCAGGACCC	TAGTAAGCGTGCTCCCACAC	59°C

**Table 3:** Primary antibodies used for Western blot analyses in murine brain tissue.

Name	Manufacturer information	Dilution
ADAM10	AB-19026, Merck Millipore	1 : 500
BACE1	AB-5832, Merck Millipore	1 : 400
PGC1	sc-5816, Santa Cruz Biotechnology	1 : 200
TFAM	sc-166965, Santa Cruz Biotechnology	1 : 200

**Table 4:** Feed intake [g], final body weight [g], and energy expenditure [kJ/(h∗kg^0.75^)] in mice fed a control diet or a diet supplemented with tocotrienol/*γ*-cyclodextrin (T3CD).

	Control	T3CD
Feed intake [g/d]	2.91 ± 0.04	2.94 ± 0.03
Final body weight [g]	41.2 ± 2.11	44.5 ± 1.65
Energy expenditure [kJ/(h∗kg^0.75^)]	22.9 ± 0.46	22.3 ± 0.47

Values are means ± SEM.

## Abbreviations

ADAM: A disintegrin and metalloproteinase domain-containing protein 10
ATP: Adenosine triphosphate
BACE1: Beta secretase 1
Cat: Catalase
CD: Cyclodextrin
Gclm: Glutamate-cysteine ligase, modifier subunit
Gpx4: Glutathione peroxidase 4
Hmox1: Heme oxygenase 1
MMP: Mitochondrial membrane potential
mtDNA: Mitochondrial DNA
Nrf2: Nuclear factor erythroid-like 2 derived factor 2
PGC1: Peroxisome proliferator-activated receptor *γ* (PPAR*γ*)
Sod2: Superoxide dismutase 2
T3: Tocotrienol
T3CD: Tocotrienol/*γ*-cyclodextrin
TFAM: Transcription factor A, mitochondrial.
